# China Stroke Registry for Patients With Traditional Chinese Medicine (CASES-TCM): Rationale and Design of a Prospective, Multicenter, Observational Study

**DOI:** 10.3389/fphar.2021.743883

**Published:** 2021-08-31

**Authors:** Luda Feng, Lingbo Kong, Xinglu Dong, Xinxing Lai, Dandan Zhang, Beida Ren, Shen Liu, Xiaolong Xie, Chuanpeng Li, Yuebo Song, Yawei Du, Kegang Cao, Chi Zhang, Ying Gao, On Behalf of the CASES-TCM Protocol Steering Group

**Affiliations:** ^1^ Department of Neurology, Dongzhimen Hospital, Beijing University of Chinese Medicine, Beijing, China; ^2^ Institute for Brain Disorders, Beijing University of Chinese Medicine, Beijing, China; ^3^ Dongzhimen Hospital, Beijing University of Chinese Medicine, Beijing, China; ^4^ Chinese Medicine Key Research Room of Brain Disorders Syndrome and Treatment of the National Administration of Traditional Chinese Medicine, Beijing, China

**Keywords:** stroke registry, acute ischemic stroke, acute intracerebral hemorrhage, traditional Chinese medicine, real-world setting, Chinese population

## Abstract

**Background:** Given the complexity of stroke treatment and the current widespread use of traditional Chinese medicine (TCM) in the absence of robust, large, long-term effectiveness and safety studies, and the lack of nationwide epidemiology and clinical characteristics of patients with stroke receiving TCM treatment, the acquisition of data from longitudinal cohorts is essential. We intend to generate the major clinical characteristics of patients with stroke who receive TCM treatment and to investigate the effectiveness and safety of TCM in the Chinese population.

**Methods:** The China Stroke Registry for Patients with Traditional Chinese Medicine (CASES-TCM) study is a prospective, multicenter, observational disease registry aiming to register 20,000 hospitalized patients. Eligible adult patients with clearly diagnosed acute ischemic stroke or intracerebral hemorrhage within 7 days of symptom onset will be consecutively registered from 126 participating sites across China. Baseline data will be recorded, and all patients will be regularly followed up at 3, 6, 12, and 24 months after stroke onset. Collected data will be entered into a web-based system with high-level data security. The primary outcomes include the distribution of scores on the modified Rankin Scale at the 3-months follow-up, and recurrent stroke events within the 12-months follow-up.

**Conclusion:** To our knowledge, the CASES-TCM study is the first and largest nationwide registry to document comprehensive data on TCM treatment in patients with acute stroke. The findings of this study will be valuable to improve our knowledge about TCM treatment for patients with stroke and its subsequent outcomes in the actual clinical setting, consequently facilitating and standardizing the optimization of individualized interventions with TCM for stroke prevention and treatment in China.

**Study registration:** This study was registered with Clinicaltrials.gov (URL: https://clinicaltrials.gov/, Unique identifier: NCT04921397).

## Introduction

Stroke is the second leading cause of death globally, and it ranks first in China, in which the annual stroke mortality rate is approximately 114.8/100,000, which is five times that of American and European countries (Wang et al., 2017; GBD 2019 Diseases and Injuries Collaborators, 2020; Zhou et al., 2019). Stroke poses a heavy health and economic burden, which is expected to increase further as a result of population aging (Rajsic et al., 2019). The distribution of stroke burden varies among different regions in China (Wang et al., 2017). Ischemic stroke and intracerebral hemorrhage (ICH) are the two most common subtypes of stroke with a total proportion of roughly 90% (Wang et al., 2011).

The current status of stroke treatment remains unoptimistic, although substantial progress has been achieved. Reperfusion therapy has been recommended as the first-line treatment of acute ischemic stroke (AIS) for improving long-term functional outcome, but it only benefits a limited number of patients given the narrow time-window, imaging dependence, high technical requirements, advanced catheter systems, and requirement for extensive economic resources (Powers et al., 2015; Powers et al., 2019). Although antiplatelet, lipid-lowering, and antihypertensive therapy carry remarkable effects for the secondary prevention of stoke (Wang et al., 2016a), more than half of the Chinese population bear aspirin or clopidogrel resistance (Wang et al., 2016b; Yi et al., 2016), and poor long-term medication compliance merits attention (Wei et al., 2010; Chen et al., 2014). As for ICH, there are no specific treatment options for patients who are ineligible for surgery (Hemphill et al., 2015). Consequently, traditional Chinese medicine (TCM) therapy, as an important, and alternative approach to the treatment of stroke, warrants investigation.

TCM has been applied in stroke treatment for thousands of years in China (Chan, 2014). TCM is popularized with wide acceptability, and results of the China Quality Evaluation of Stroke Care and Treatment study, which examined the clinical practice and secondary prevention of stroke in China, indicated that TCM accounted for 83.1% of AIS medication treatment and 42% of ICH treatment (Group of the China Quality Evaluation of Stroke Care and Treatment, 2009; Wei et al., 2011). Additionally, deemed as a part of TCM, acupuncture for stroke treatment has been used in China for over 1,000 years and is increasingly practiced in some western countries (Wu et al., 2006; Zhang et al., 2015).

However, the epidemiology and clinical characteristics of patients with stroke receiving TCM treatment remain unknown, and the actual effects concerning benefits and harms of TCM for stroke are still conflictive. Some of the therapeutic effects of TCM on stroke treatment have been proven by previous clinical trials in terms of alleviating neurological impairment (Gao L et al., 2012), improving long-term physical disability and handicap (Hu et al., 2020), facilitating limb, language and dysphagia recovery (Liu and Zhang, 2006; Zhang et al., 2013; Xia et al., 2016; Yu et al., 2021), accelerating hematoma absorption (Gao Y et al., 2012; Li et al., 2016), and reducing recurrent stroke events (Chen et al., 2013a; Cai et al., 2019). These trials were conducted at ideal conditions without multiple confounding factors, indicating that external application of TCM treatment requires validation under updated clinical practice. Moreover, no evidence of comparative effectiveness is available from cohorts of patients treated outside the context of clinical trials. By contrast, several trials demonstrated that TCM neither improved long-term functional outcome of AIS nor reduced hematoma enlargement in ICH (Chen et al., 2013b; Zeng et al., 2019). The possible reasons may lie in similar reflection on the failure of past trials, which includes wrong timing or wrong dosage of TCM administration, inadequate sample size, inaccurate target population, and inter-center variations in treatment (Deans et al., 2007; Vincent, 2010; Macdonald et al., 2013). Additionally, as one of the largest cohort studies regarding acute cerebrovascular events in previous decades in China, the China National Stroke Registry (CNSR) demonstrated that the wide usage of TCM treatment might be associated with higher stroke recurrence (Qin et al., 2021). However, it is notable that the CNSR did not report detailed TCM treatment information but only roughly recorded TCM as an exposure.

Considering the importance of clinical data on stroke in the Chinese population treated with TCM in the real world, and to help fill the clinical gap, we therefore designed the China Stroke Registry for Patients with Traditional Chinese Medicine (CASES-TCM) study to generate sociodemographic and medical data relating to patients with stroke receiving TCM treatment and to investigate the effectiveness and safety of TCM for stroke in the Chinese population.

## Methods and Design

### Study Design

**FIGURE 1:**
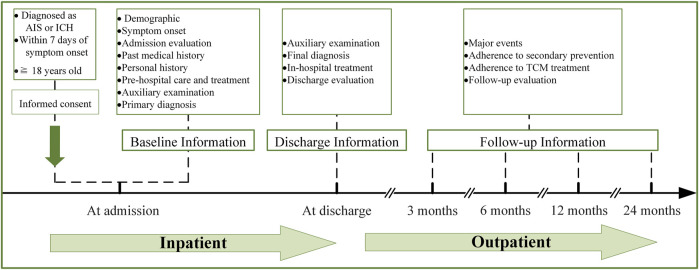
The flowchart of the CASES-TCM study; Abbreviations: AIS, acute ischemic stroke; ICH, intracerebral hemorrhage; TCM, traditional Chinese medicine.

The CASES-TCM study (registered with ClinicalTrials.gov, Unique identifier: NCT04921397) is a prospective, multicenter, observational disease registry, attempting to depict major clinical characteristics of stroke in patients treated with TCM and to explore any difference in the comparison with other non-TCM user cohorts and the effectiveness and safety of TCM. An overview of this study flowchart is shown in Figure 1. The registry protocol was approved by the institutional review board of the leading center, Dongzhimen Hospital, Beijing University of Chinese Medicine, Beijing, China (No. 2021DZMEC-024-02), and will be approved by the local institutional review boards of all participating sites.

### Patient Population

Adult patients (≥18 years) admitted to Chinese hospitals with AIS or ICH diagnosed according to the corresponding guidelines, respectively, are eligible for inclusion in the CASES-TCM study (Hemphill et al., 2015; Powers et al., 2019). Only patients within 7 days of symptom onset will be included. Patients who have difficulties with follow-up completion will be excluded. Written informed consent including participation, data collection, and publication will be obtained from all patients or their legally authorized representatives in this study. Patient participation will be entirely voluntary, and patient’s data will be strictly protected. There is no specific risk of participation in this study. Instead, patients will receive regular medical guidance from doctors during the follow-up period. Patient registry is expected to begin in September 2021.

### Specific Treatment

Different kinds of TCM treatment for patients with stroke will be included because we will focus on whether doctors use TCM based on individual syndrome differentiation, which is etiology in view of TCM theory. We will record TCM application settings including prehospital stage (at home or ambulance), in-hospital stage (emergency department or neurology ward), and hospital discharge (community or at home), and TCM formulations including Chinese patent medicine (injection or oral agents), and TCM compounds. The initiation time of TCM administration for patients with stroke and adherence to TCM treatment will also be recorded. As for other routine treatments, doctors will treat patients with stroke according to the corresponding Chinese guidelines (Chinese Society of Neurology, 2018; Chinese Society of Neurology, 2019). The common management methods include basic control of risk factors, intravenous thrombolysis, mechanical thrombectomy, antiplatelets, anticoagulants, and neuroprotective agents. Additionally, doctors will determine the treatment strategies based on their clinical experience and the patients’ condition when no guideline-recommended therapies are applicable.

### Main Variables

We established the minimum dataset that includes the main variables using a stepwise approach. To ensure the balance between feasibility and scientificity, we pooled the variables from our previous studies (ChiCTR1800016363, NCT02728180, ChiCTR-OPC-16008451, ChiCTR1900026422) on stroke survey and treatment, which were funded by the Stroke Prevention Project Committee National Health and Family Planning Commission, and the Ministry of Science and Technology of the People’s Republic of China. Taking into account the TCM treatment features, patient burden, potential loss to follow-up, and comparability to variables of other representative large-scale registry studies, we adjusted the variables and finally determined the minimum dataset *via* expert consensus. The expert panel consisted of clinicians, researchers, healthcare nurses, and experts from other disease registries. Table 1 lists the main variables that will be collected during the study.

**TABLE 1:** Main variables registered in CASES-TCM study.

Field	Variables
**Basic Information**	
Demographic	Age; Gender; Ethnicity; Education; Birthplace; Permanent address; Height; Weight; Body Mass Index; Marital status; Pregnancy; Occupation; Medical insurance
**Baseline Information (At admission)**	
Symptom onset	Symptom onset date; Solar terms; Five evolutive phases and six climatic factors (*Wuyun Liuqi*); Admission date; Pre-onset transient symptoms; Pre-onset mRS; Condition at onset; Initial symptoms
Admission evaluation	NIHSS; GCS; mRS; BI; MMSE; DSSEIS
Past medical history	Cerebrovascular diseases; Heart diseases; Hypertension; Diabetes mellitus; Dyslipidemia; Peripheral arterial disease; Hyperuricemia; Hyperhomocysteinemia; Others
Personal history	Smoking; Alcohol consumption; Lifestyle (diet preference; exercise frequency)
Pre-hospital care and treatment/Previous medication	First aid transport mode; Reperfusion therapies (IVT; EVT) for AIS; Surgery for ICH; Antiplatelet agents; Anticoagulants; Neuroprotective agents; TCM (specific medicine; acupuncture; application setting), Others
Auxiliary examination	Vital signs; Laboratory tests completed within 24 h; ECG; Neuroimaging; Examination for other risk factors
Primary diagnosis	AIS or ICH; Chinese Medicine Diagnosis; OCSP classification of AIS/Lesion classification of ICH
**Discharge Information**	
Auxiliary examination	Vital signs; Laboratory tests
Final diagnosis	TOAST classification of AIS; SMASH-U classification of ICH; In-hospital complications
In-hospital treatment	Reperfusion therapies (IVT; EVT) for AIS; Surgery for ICH; Antiplatelet agents; Anticoagulants; Lipid-lowering agents; Antihypertensive agents; Hypoglycemic agents; Neuroprotective agents; TCM (Chinese patent medicine; TCM compounds; acupuncture; others): including administration time and dose
Discharge evaluation	NIHSS; GCS; mRS; BI; MMSE; DSSEIS; PRO-Stroke
**Follow-up Information**	
Major events	Death; New cardiovascular events; New cerebrovascular events; Adverse events
Adherence to secondary prevention	Antiplatelet agents; Anticoagulants; Lipid-lowering agents; Antihypertensive agents; Hypoglycemic agents
Adherence to TCM treatment	Specific medicine; Proportion of days covered of TCM treatment
Follow-up evaluation	mRS; BI; MMSE

mRS, modified Rankin Scale; NIHSS, National Institutes of Health Stroke Scale; BI, Barthel Index; MMSE, Mini-Mental State Examination; DSSEIS, Diagnostic Scale of Syndrome Elements in Ischemic Stroke; IVT, Intravenous thrombolytic therapy; EVT, endovascular treatment; AIS, acute ischemic stroke; ICH, intracerebral hemorrhage; TCM, traditional Chinese medicine; ECG, electrocardiogram; OCSP, Oxfordshire Community Stroke Project; TOAST, Trial of Org 10172 in Acute Stroke Treatment; SMASH-U, Structural lesion, Medication, Amyloid angiopathy, Systemic/other disease, Hypertension, Undetermined; PRO-Stroke, Patient-Reported Outcome Scale for Stroke.

### Data Collection and Monitoring

During patient admission, trained research personnel who are appointed at each participating site will collect baseline data regarding prehospital care and treatment, pre-stroke symptom, and the pre-stroke modified Rankin Scale (mRS) score and will perform assessments using the Glasgow Coma Scale (GCS), National Institute of Health Stroke Scale (NIHSS), Barthel Index (BI), Mini-Mental State Examination (MMSE), and Diagnostic Scale of Syndrome Elements in Ischemic Stroke (DSSEIS). The patients’ demographics, medical history, family history, physical examination, auxiliary examination, risk factor assessment, and clinical diagnosis will be extracted from medical records. Research personnel will perform GCS, NIHSS, mRS, BI, MMSE, DSSEIS, and Patient-Reported Outcome Scale for Stroke (PRO-Stroke) assessment through a face-to-face interview during patient discharge. The etiologic classification of stroke, stroke-related complications, and new cerebrovascular events will be extracted. The etiologic classification of AIS will be determined according to the Trial of Org 10172 in Acute Stroke Treatment (TOAST) criteria while ICH according to the Structural lesion, Medication, Amyloid angiopathy, Systemic/other disease, Hypertension, Undetermined (SMASH-U) criteria (Adams et al., 1993; Meretoja et al., 2012). In addition to conventional therapies, research personnel will record the type of TCM treatment that is defined according to the World Health Organization (World Health Organization, 2011), details involving Chinese patent medicine, TCM compounds, hospital preparation of TCM, acupuncture, and moxibustion during hospitalization. Data collection and reporting will be conducted using an electronic data capture (EDC) system (http://www.1-dao.net:13579/) with a unique ID. Research personnel can also launch this EDC system on smartphones, and they will complete data collection when they are on daily ward rounds. All laboratory test and auxiliary examination results will be collected. The EDC system can remind research personnel of patients’ follow-up, and it can also automatically check for completeness, coding, value range, and logical error of uploaded data, and then provide feedback to research personnel. Any uploaded data correction, as well as electronic audit trail with electronic signature and date, will be recorded on the system. This registry system is housed on a secure server at the Institute for Brain Disorders, Beijing University of Chinese Medicine. An independent contract research organization will perform online data monitoring during the whole study.

### Follow-Up Procedures

All patients will be identified and followed up using individual identity cards that are unique to every Chinese citizen, enabling unambiguous linkages. Research personnel will regularly perform face-to-face or telephone follow-up at 3, 6, 12, and 24 months after stroke onset. Information, including death, functional status, activity of daily living, cardiovascular or cerebrovascular events, cognitive function, and adherence to secondary prevention recommendation and TCM treatments, will be obtained. Death will be confirmed *via* a death certificate from the local civil registry or from the attended hospital. New cardiovascular or cerebrovascular events leading to rehospitalization will be confirmed by the discharge diagnosis. Suspected recurrent cardiovascular or cerebrovascular events will be determined by the endpoint judgment committee if without hospitalization (Wang et al., 2019).

### Study Outcomes

In the CASES-TCM study, the distribution of scores on the mRS at the 3-months follow-up, and any recurrent stroke events including ischemic and hemorrhagic stroke (ICH and subarachnoid hemorrhage) within the 12-months follow-up will be set as the primary outcomes. Secondary outcomes, which will be assessed at different follow-up visits, include the following: exposure and adherence to TCM treatment, improvement of neurological deficits, patients’ subjective feelings, death, functional outcome, activity of daily living, cognitive function, composite of new clinical vascular events, and incidence of adverse events. The complete outcomes of the CASES-TCM study are illustrated in Table 2.

**TABLE 2:** The complete outcomes of CASES-TCM study.

**Primary outcomes**
Distribution of scores on the mRS at 3-months follow-up
Recurrent stroke events (ischemic stroke, hemorrhagic stroke including intracerebral hemorrhage and subarachnoid hemorrhage) within 12-months follow-up
**Secondary outcomes**
Proportion of patients receive TCM treatment within 24 h after admission
TCM treatment duration within 3-months, 12-months, and 24-months follow-up
Change in the NIHSS score between baseline (admission) and discharge (14 days—mean for length of hospitalization for stroke patients)
Patient-Reported Outcomes Scale for Stroke score at discharge (14 days—mean for length of hospitalization for stroke patients)
Proportion of patients with mRS score ≤2 at 3-months and 12-months follow-up
Distribution of scores on the mRS at 12-months follow-up
Proportion of patients with BI score ≥90 at 3-months and 12-months follow-up
Recurrent stroke events (ischemic stroke, hemorrhagic stroke including intracerebral hemorrhage and subarachnoid hemorrhage) within 3-months and 24-months follow-up
Composite of new clinical vascular events within 3-months, 12-months, and 24-months follow-up
MMSE scale score at 3-months, 12-months, and 24-months follow-up
All-cause mortality within 12-months and 24-months follow-up
Incidence of adverse events during the whole study

mRS, modified Rankin scale; TCM, traditional Chinese medicine; NIHSS, National Institute of Health Stroke Scale; BI, Barthel Index; MMSE, Mini-Mental State Examination.

### Study Sites and Data Source

**FIGURE 2:**
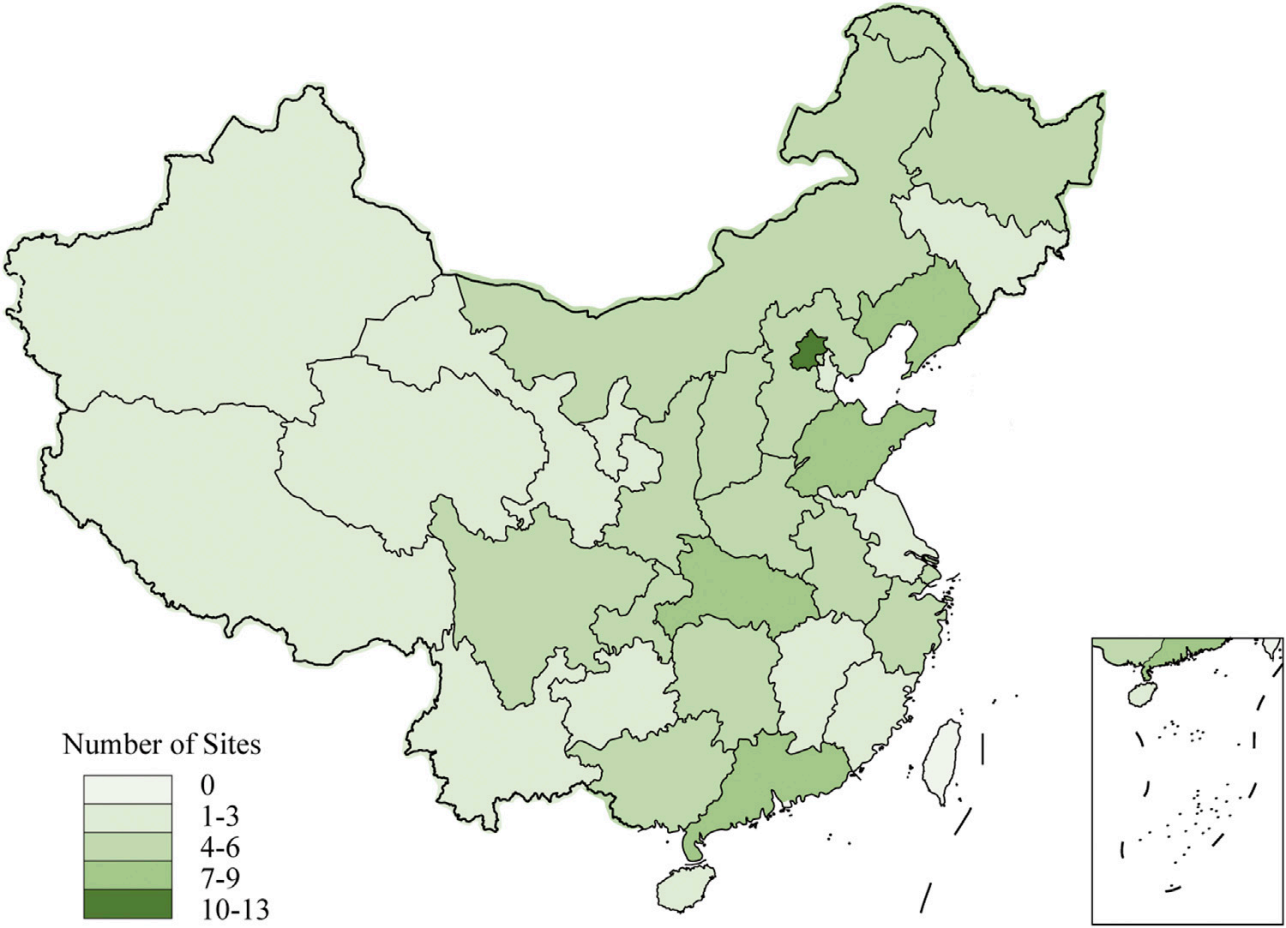
Sites distribution of CASES-TCM study.

The steering committee of the CASES-TCM study will screen hospitals nationwide from each region in the north, east, west, south, and center of mainland China to represent the population. Participating sites in our study are required to admit over 100 cases of patients with stroke per year, have experienced multicenter studies, and be equipped with magnetic resonance imaging or computed tomography machines. A total of 126 participating sites with qualified research capability and proven commitment to the study will be ultimately selected. Figure 2 illustrates the geographical distribution of the selected participating sites.

### Quality Control and Management

Prior to study initiation, the steering committee will create training videos regarding the study protocol and standard operating procedures (SOPs) in terms of consecutive patient screening and recruitment, obtaining informed consent, and data collection. Research personnel will be required to complete the training videos and obtain a study permit once they pass the examination. The endpoint judgment committee will be responsible for developing SOPs concerning the proper use of scales, outcome assessment, and reporting procedures. An independent data safety and monitoring board will conduct data monitoring to ensure adherence to study documentation, reporting procedures, and the study protocol.

### Study Sample Size

Generally speaking, the sample size of the registry study should be sufficiently large enough to represent the general population. Based on the annual number of 20 cases of hospitalization per month due to acute stroke in every participating site (a total of 126 sites), we aim to register 20,000 patients in the CASES-TCM study. Enrolment of up to 20,000 patients is anticipated at the end of the first year.

### Statistical Analysis

A statistical analysis plan (SAP) has been developed prior to the initiation of the CASES-TCM registry. All collected observational data will be analyzed descriptively using established statistical methods. The mean, median, standard median, or interquartile ranges will be used to summarized continuous data, while counts or percentages for categorical data. Between-group comparisons will be conducted using parametric tests [one-way analysis of variance (ANOVA) or Student’s t-test] or non-parametric tests (chi-square test, Fisher’s exact test, Kruskal–Wallis one-way ANOVA on ranks, or Mann–Whitney *U* test), as appropriate. As for time-to-event analysis, patients will be censored at their last follow-up assessment when experiencing a clinical event, at the end of study, or at the time of withdrawal from the study. Cumulative clinical events will be reported as Kaplan–Meier estimates. Cox proportional hazards regression methods will be used for hazard ratio calculation at 95% confidence intervals, and the treatment effect will be assessed using the log-rank test. During the analysis, confounding factors will be considered, and multivariable regressions will be conducted with adjustments for potential covariates and the propensity score. If not specified otherwise in the SAP, missing data will not be imputed. Subjects with incomplete data will be included in the analysis and for each variable; the number of subjects with missing data will be reported. Interim analyses comprising descriptive summaries of collected data will be conducted every 6 months. All statistical tests will be two-sided and statistical significance will be set at *p* < 0.05. Statistical analyses will be performed using SAS software version 9.4 (SAS Institute, Inc.).

## Discussion

Due to the emphasis on the effectiveness, benefits, and disadvantages of various treatment methods, well-designed large-scale registries for patients with stroke that are linked with long-term clinical outcomes should be a priority for clinicians and researchers (Kim et al., 2014). We describe the rationale and design of a prospective, multicenter, registry-based observational study, which will investigate the clinical features, stroke management with TCM, and outcomes of patients with acute stroke in Chinese real-world settings. Given that current guideline-recommended evidence-based treatments for AIS and ICH are limited, there remains a need for an effective and safe therapy for improving the prognosis of patients with stroke. TCM treatments are commonly used for stroke in China but the evidence is insufficient (Wu et al., 2007; Wu et al., 2019). Therefore, the practical effects and disadvantages of TCM in patients with stroke need to be confirmed *via* a large longitudinal data collection. This study will provide information on potential beneficiary from TCM, proper time to initiate TCM, and appropriate duration of TCM treatment for patients with stroke, on the basis of updated guideline-recommended routine treatment. Besides, this registry-based observational study could reveal the treatment patterns, such as effectiveness of specific treatment, switching therapies during follow-up, and concomitant medications in a real-world setting, which are unlikely to be obtained in conventional clinical trials (Sherman et al., 2016).

Proper TCM use is based on its unique theory system including holism and syndrome differentiation. The core concept of TCM syndrome differentiation lies in personalized treatment (Yuan, 2021). Complex and diversified information are needed to guide personalized treatment to maximize the patient benefits. We will not only examine the natural history of stroke but also focus more on the effects of TCM treatment for stroke based on the TCM theory system in the present study. Table 3 lists the comparison of our study with the other two stroke registries with available TCM information, including study objectives, target population, study scale, area coverage, and disease subtypes (Hao et al., 2011; Wang et al., 2011; Qin et al., 2021).

**TABLE 3:** Comparison of CSR, CNSR, and CASES-TCM study.

	CSR Hao et al. (2011)	CNSR Wang et al. (2011)	CASES-TCM
Registration number	None	None	NCT04921397
Study year	2002–2006	2007–2008	2021
Publish year	2011	2011	-
Times cited	139	215	-
Study objectives	To analyze basic data and outcomes in Chengdu Stroke Registry	To evaluate the quality of care for stroke patients in China	To depict major clinical characteristics of stroke in patients treated with TCM and to explore any difference in the comparison with other non-TCM user cohorts and the effectiveness and safety of TCM.
Study region	West China	Across China	Across China
Number of sites	1	132	126
Number of patients	3,123	21,902	Anticipated 20,000
Onset to admission	≤30 days	≤14 days	≤ 7 days
Disease subtypes	AIS, TIA, ICH, SAH	AIS, TIA, ICH, SAH	AIS, ICH
Follow-up	12 months	24 months	24 months
TCM information	✓ Only report TCM treatment as an exposure	✓ Only report TCM treatment as an exposure Qin et al. (2021)	✓ Pre-hospital TCM treatment (specific medicine; application setting)
	✓ Inadequately describe important aspects of TCM use	✓ Inadequately describe important aspects of TCM use	✓ In-hospital TCM treatment (Chinese patent medicine; TCM compounds; acupuncture; others): including administration time and dose
			✓ Adherence to TCM treatment

CSR, Chengdu Stroke Registry; CNSR, China National Stroke Registry; CASES-TCM, China Stroke Registry for Patients with Traditional Chinese Medicine; TCM, traditional Chinese medicine; AIS, acute ischemic stroke; TIA, transient ischemic attack; ICH, intracerebral hemorrhage; SAH, subarachnoid hemorrhage.

Similar to other registry studies, the presence of selection bias of participating sites in the CASES-TCM study is unavoidable although the sample population covers most areas of China (Ohman et al., 2006; Wang et al., 2011). However, the multicenter participation and regular follow-up of consecutively enrolled hospitalized patients in this large-scale clinical-based study guarantee the cross-sectional and longitudinal data. We could explore both the geographical and temporal characteristics of patients with stroke in China to improve the representation of collected data in terms of a disease registry. Further, we will conduct strict quality control with respect to participating site screening, research personnel training, and timely data audit to ensure the accuracy and completeness of collected data. Moreover, some studies with specific hypotheses such as comparative effectiveness research, pragmatic randomized clinical trial, or nested case-control study can also be embedded within this registry study, thereby generating secondary hypotheses for further validation in clinical trials (Lei et al., 2020).

To the best of our knowledge, the CASES-TCM study is the first and largest, comprehensive, prospective, registry-based, observational study to report TCM application for stroke in China, which investigates the major clinical characteristics of stroke in patients treated with TCM and to explore any difference in the comparison with other non-TCM user cohorts and the effectiveness and safety of TCM in real-world settings. As an important information source and quality improvement instrument for stroke care in China, the CASES-TCM study will improve our knowledge about clinical management with TCM for patients with stroke and its subsequent outcomes under the influence of other confounding factors. The results will eventually facilitate and standardize the optimization of individualized interventions with TCM for stroke prevention and treatment in China.

## Abbreviations

AIS, acute ischemic stroke; ANOVA, analysis of variance; BI, Barthel Index; CASES-TCM, China Stroke Registry for Patients with Traditional Chinese Medicine; CNSR, China National Stroke Registry; DSSEIS, Diagnostic Scale of Syndrome Elements in Ischemic Stroke; EDC, electronic data capture; GCS, Glasgow Coma Scale; ICH, intracerebral hemorrhage; MMSE, Mini-Mental State Examination; mRS, modified Rankin Scale; NIHSS, National Institute of Health Stroke Scale; PRO-Stoke, Patient-Reported Outcome Scale for Stroke; SAP, statistical analysis plan; SMASH-U, Structural lesion, Medication, Amyloid angiopathy, Systemic/other disease, Hypertension, Undetermined; SOP, standard operating procedures; TCM, traditional Chinese medicine; TOAST, Trial of Org 10172 in Acute Stroke Treatment.
