# Supplementary material for: China Stroke Registry for Patients With Traditional Chinese Medicine (CASES-TCM): Rationale and Design of a Prospective, Multicenter, Observational Study
Source: Front Pharmacol. 2021 Aug 31;12:743883. doi: 10.3389/fphar.2021.743883 (PMC8438566; doi:10.3389/fphar.2021.743883)
Supplement: Supplementary file 1 [file Table1.pdf]

Supplementary file

List of participating sites in the CASES-TCM study.

**Province, Autonomous Region, and Special Administrative Region**	**Number of sites**	**Participating sites**
Beijing	13	Dongzhimen Hospital, Beijing University of Chinese Medicine (Tongzhou District)
		Dongzhimen Hospital, Beijing University of Chinese Medicine
		Beijing University of Chinese Medicine Third Affiliated Hospital
		Xuanwu Hospital affiliated to Capital Medical University
		Dongfang Hospital, Beijing University of Chinese Medicine
		Beijing Mentougou Hospital of Traditional Chinese Medicine
		Beijing Tibetan Hospital
		Fangshan Hospital, Beijing University of Chinese Medicine
		Shunyi Hospital, Beijing Traditional Chinese Medicine Hospital
		Beijing Huairou Hospital of Traditional Chinese Medicine
		Beijing First Hospital of Integrated Traditional Chinese and Western Medicine
		Beijing Hepingli Hospital
		Beijing Chaoyang Integrative Medicine Emergency Medical Center
Liaoning	8	The Second Affiliated Hospital of Liaoning University of Traditional Chinese Medicine
		Anshan Hospital of Traditional Chinese Medicine
		Dalian Hospital of Integrated Traditional Chinese and Western Medicine
		Dalian Hospital of Traditional Chinese Medicine
		Dandonog Hospital of Traditional Chinese Medicine
		Yingkou Hospital of Traditional Chinese Medicine
		Sujiatun Hospital of Traditional Chinese Medicine
		Benxi Center Hospital
Shandong	8	The Second Affiliated Hospital of Shandong University of Traditional Chinese Medicine

		Shandong Provincial Qianfoshan Hospital
		Tai'an Hospital of Traditional Chinese Medicine
		Weifang Hospital of Traditional Chinese Medicine
		Rizhao Hospital of Traditional Chinese Medicine
		Zibo Hospital of Traditional Chinese Medicine
		Yantai Yuhuangding Hospital
		Affiliated Hospital of Shandong University of Traditional Chinese Medicine
Guangdong	7	Nanfang Hospital, Southern Medical University
		Foshan Hospital of Traditional Chinese Medicine
		Wuyi Hospital of Traditional Chinese Medicine
		Guangdong Province Traditional Chinese Medical Hospital
		The First Affiliated Hospital of Guangzhou University of Chinese Medicine
		The Affiliated Traditional Chinese Medicine Hospital of Guangzhou Medical University
		Shenzhen Hospital, Beijing University of Chinese Medicine
Hubei	7	Hubei Provincial Hospital of Traditioanl Chinese Medicine
		Ezhou Hospital of Traditional Chinese Medicine
		Wuhan Hospital of Traditional Chinese and Western Medicine
		Wuhan Hospital of Traditional Chinese Medicine
		Xiangyang Hospital of Traditional Chinese Medicine
		Renmin Hospital of Wuhan University
		Hubei Provincial Hospital of Integrated Chinese and Western Medicine
Hebei	6	Handan Hospital of Traditional Chinese Medicine
		Hebei Yiling Hospital
		Traditional Chinese Medicine Hospital of Shijiazhuang City
		Qian'an City Hospital of Traditional Chinese Medicine
		Tangshan Hospital of Traditional Chinese Medicine
		Qinhuangdao Hospital of Traditional Chinese Medicine
Henan	6	The first Affiliated Hospital of Henan University of Chinese Medicine
		Henan Province Hospital of Traditional Chinese Medicine
		Luoyang Second Hospital of Traditional Chinese Medicine

		Zhengzhou Traditional Chinese Medicine Hospital
		Zhumadian Traditional Chinese Medicine Hospital
		Jiaozuo Hospital of Traditional Chinese Medicine
Guangxi	6	The First Affiliated Hospital of Guangxi University of Chinese Medicine
		Ruikang Hospital Affiliated to Guangxi University of Chinese Medicine
		The First Affiliated Hospital of Guangxi Medical University
		Traditional Chinese Medicine Hospital of Yulin
		Liuzhou Tradtional Chinese Medical Hospital
		Nanning Hospital of Traditional Chinese Medicine
Sichuan	5	Hospital (T.C.M.) Affiliated to Southwest Medical University
		Chengdu University of Traditional Chinese Medicine Affiliated Hospital
		Chengdu Intergrated Traditional Chinese Medicine and Western Medicine Hospital
		Luzhou Hospital of Traditional Chinese Medicine
		Panzhihua Center Hospital
Shaanxi	5	Shaanxi Provincial Hospital of Chinese Medicine
		Affiliated Hospital of Shaanxi University of Chinese Medicine
		Xi'an Hospital of Traditional Chinese Medicine
		Xi'an Traditional Chinese Medicine Brain Disease Hospital
		Ankang Hospital of Traditional Chinese Medicine
Shanghai	5	Zhongshan Hospital, Fudan University
		Shanghai Hospital of Traditional Chinese Medicine
		Shanghai Putuo District Hospital of Traditional Chinese Medicine
		Shanghai Xuhui District Hospital of Traditional Chinese Medicine
		Shuguang Hospital, Shanghai University of Traditional Chinese Medicine
Chongqing	5	Chongqing Traditional Chinese Medicine Hospital
		Beipei Traditional Chinese Medical Hospital
		Fuling Traditional Chinese Medical Hospital
		Chongqing Hospital of Integrated Traditional Chinese and Western Medicine
		Dianjiang Hospital of Traditional Chinese Medicine
Anhui	4	The First Affiliated Hospital of Anhui University of Chinese Medicine
		Chuzhou Hospital of Integrated Traditional Chinese and Western Medicine

		Guoyang Hospital of Traditional Chinese Medicine
		The Second Affiliated Hospital of Anhui University of Chinese Medicine
Hunan	4	Xiangya Hospital Central South University
		The Second Hospital of Hunan University of Chinese Medicine
		Hunan Academy of Traditional Chinese Medicine Affiliated Hospital
		Liling Hospital of Traditional Chinese Medicine
Zhejiang	4	Zhejiang Provincial Hospital of Chinese Medicine
		Yiwu Hospital of Traditional Chinese Medicine
		Zhejiang Geriatric Care Hospital
		Tongde Hospital of Zhejiang Province
Shanxi	4	The Third Traditional Chinese Medicine Hospital of Shanxi Unversity of Chinese Medicine
		Integrated Traditional Chinese and Western Medicine Affiliated to Shanxi Unversity of Chinese Medicine
		Shanxi University of Chinese Medicine Affiliated Hospital
		Affiliated Hospital of Changzhi Institute of Traditional Chinese Medicine
Inner Mongolia	4	Hulun Buir Hospital of Traditional Chinese and Inner Mongolian Medicine
		Inner Mongolia International Mongolian Hospital
		Inner Mongolia Hospital of Traditional Chinese Medicine
		Hohhot Hospital of Inner Mongolian Medicine and Traditional Chinese Medicine
Heilongjiang	4	The Second Affiliated Hospital of Heilongjiang University of Chinese Medicine
		Harbin Traditonal Chinese Medicine Hospital
		Jixi Hospital of Traditional Chinese Medicine
		The First Affiliated Hospital of Heilongjiang University of Chinese Medicine
Tianjin	3	Second Affiliated Hospital of Tianjin University of Traditional Chinese Medicine
		Tianjin Hospital of ITCWM Nankai Hospital
		Wuqing Traditional Chinese Medicine Hospital Affiliated to Tianjin University of Traditional Chinese Medicine
		Lianyungang Hospital of Traditional Chinese Medicine

Jiangsu	3	Nanjing Hospital of Chinese Medicine Affiliated to Nanjing University of Chinese Medicine
		Taizhou Hospital of Traditional Chinese Medicine
Jilin	2	Changchun University of Chinese Medicine Affiliated Hospital
		Yanji Hospital of Traditional Chinese Medicine
Jiangxi	2	Nanchang Hongdu Hospital of Traditional Chinese Medicine
		Jiujiang Hospital of Traditional Chinese Medicine
Gansu	2	Gansu Provincial Hospital of Traditional Chinese Medicine
		Tianshui Hospital of Traditional Chinese Medicine
Fujian	2	Quanzhou Hospital of Traditional Chinese Medicine
		Xiamen Traditional Chinese Medicine Hospital, Beijing Unversity of Chinese Medicine
Ningxia	1	Guyuan Hospital of Traditional Chinese Medicine
Hainan	1	Chinese Medicine Hospital of Hainan Province
Guizhou	1	The First Affiliated Hospital of Guizhou University of Traditional Chinese Medicine
Yunnan	1	Yunnan Provincial Hospital of Traditional Chinese Medicine
Xinjiang	1	Traditional Chinese Medicine Hospital of Xinjiang Uygur Autonomous Region
Tibet	1	Tibet Autonomous Region Tibetan Hospital
Qinghai	1	Qinghai Hospital of Traditional Chinese Medicine
Hong Kong	0	/
Macao	0	/
Taiwan	0	/
In total	126	
